# Synthesis and Evaluation of New 1,3,4-Thiadiazole Derivatives as Antinociceptive Agents

**DOI:** 10.3390/molecules21081004

**Published:** 2016-08-01

**Authors:** Mehlika Dilek Altıntop, Özgür Devrim Can, Ümide Demir Özkay, Zafer Asım Kaplancıklı

**Affiliations:** 1 Department of Pharmaceutical Chemistry, Faculty of Pharmacy, Anadolu University, Eskişehir 26470, Turkey; zakaplan@anadolu.edu.tr; 2 Department of Pharmacology, Faculty of Pharmacy, Anadolu University, Eskişehir 26470, Turkey; ozgurdt@anadolu.edu.tr (Ö.D.C.); udemir@anadolu.edu.tr (Ü.D.Ö.)

**Keywords:** activity cage, antinociceptive, hot-plate, tail-clip, thiadiazole, writhing

## Abstract

In the current work, new 1,3,4-thiadiazole derivatives were synthesized and investigated for their antinociceptive effects on nociceptive pathways of nervous system. The effects of these compounds against mechanical, thermal and chemical stimuli were evaluated by tail-clip, hot-plate and acetic acid-induced writhing tests, respectively. In addition, activity cage was performed to assess the locomotor activity of animals. The obtained data indicated that compounds **3b**, **3c**, **3d**, **3e**, **3g** and **3h** increased the reaction times of mice both in the hot-plate and tail-clip tests, indicating the centrally mediated antinociceptive activity of these compounds. Additionally, the number of writhing behavior was significantly decreased by the administration of compounds **3a**, **3c**, **3e** and **3f**, which pointed out the peripherally mediated antinociceptive activity induced by these four compounds. According to the activity cage tests, compounds **3a**, **3c** and **3f** significantly decreased both horizontal and vertical locomotor activity of mice. Antinociceptive behavior of these three compounds may be non-specific and caused by possible sedative effect or motor impairments.

## 1. Introduction

Pain, an unpleasant sensation ranging in intensity from slight through severe to indescribable, is the most common symptomatic reason to seek medical consultation. Despite the large number of currently available analgesic agents, the design of analgesic molecules for the management of pain is still the subject of ongoing research [1,2,3].

Analgesics are mainly divided into two groups: opioid analgesics and nonsteroidal anti-inflammatory drugs (NSAIDs). The fear of addiction, and tolerance related to morphine and related drugs, has resulted in the restriction and withdrawal of these drugs [1,2,3,4,5,6]. NSAIDs, which act primarily by inhibiting cyclooxygenase (COX) enzymes, are frequently prescribed drug groups to manage inflammatory pain. The long-term use of NSAIDs may lead to severe gastrointestinal side effects limiting their use. The adverse effects accompanying the use of nonselective NSAIDs arise from the reduction of the levels of protective prostaglandins in the gastrointestinal (GI) tract due to the inhibition of COX-1. Although selective COX-2 inhibitors cause less GI adverse effects than nonselective NSAIDs, their use in the treatment is also limited due to their serious cardiovascular side effects. Therefore, current research efforts are mainly directed towards the discovery of new potent analgesic drugs with fewer side effects [1,2,3,4,5,6].

Thiadiazole has attracted a great deal of interest as a privileged scaffold due to its significant therapeutic potential for central nervous system (CNS) disorders. 1,3,4-Thiadiazole derivatives have been reported to exhibit a wide range of pharmacological effects including analgesic, antidepressant, anxiolytic, and anticonvulsant activities [7,8,9,10,11,12,13,14,15,16,17,18,19,20,21]. The sulfur atom of thiadiazole ring imparts improved liposolubility, important for the drugs active at CNS level. The mesoionic nature of 1,3,4-thiadiazoles allows these compounds to cross cellular membranes and interact with biological targets with distinct affinities [7,8,9,10].

Prompted by the aforementioned findings and in continuation of our ongoing research on 1,3,4-thiadiazoles related to their antinociceptive activity [21], herein we reported the synthesis of a new series of 1,3,4-thiadiazole derivatives and focused on their antinociceptive effects.

## 2. Results

### 2.1. Chemistry

**Figure 1:**
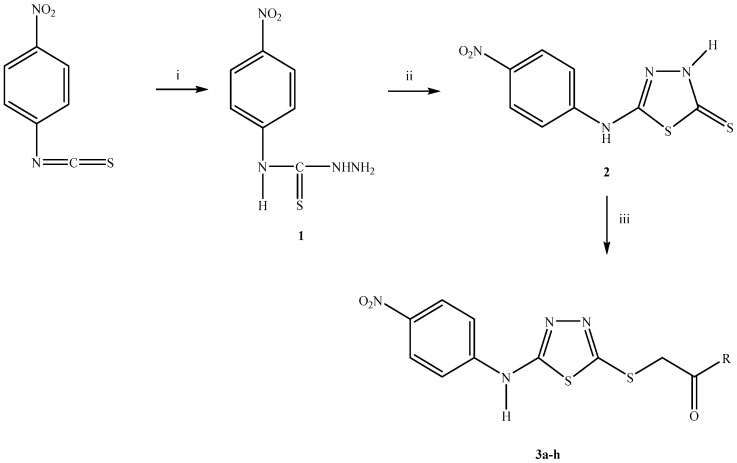
The synthetic route for the preparation of compounds **3a**–**h**. *Reagents and conditions*: (**i**) NH_2_NH_2_·H_2_O, ethanol, rt, 5 h; (**ii**) (1) CS_2_/KOH, ethanol, reflux, 10 h; (2) HCl, pH 4–5; (**iii**) ClCH_2_COR, K_2_CO_3_, acetone, rt, 8 h.

The synthesis of compounds **3a**–**h** followed the general pathway outlined in Figure 1. Initially, 4-(4-nitrophenyl)thiosemicarbazide (**1**) was obtained by the reaction of 4-nitrophenyl isothiocyanate with hydrazine hydrate. 5-(4-Nitrophenyl)amino-1,3,4-thiadiazole-2(3*H*)-thione (**2**) was synthesized via the ring closure reaction of 4-(4-nitrophenyl)thiosemicarbazide (**1**) with carbon disulphide in the presence of potassium hydroxide. Finally, the reaction of compound **2** with *N*-(alkyl/aryl)-2-chloroacetamide/4-(chloroacetyl)morpholine afforded new thiadiazole derivatives (**3a**–**h**). The yields and melting points (M.p.) of the compounds were given in Table 1. The structures of compounds **3a**–**h** were confirmed by IR, ^1^H-NMR, ^13^C-NMR, mass spectral data, and elemental analysis.

### 2.2. Pharmacology

**Figure 2:**
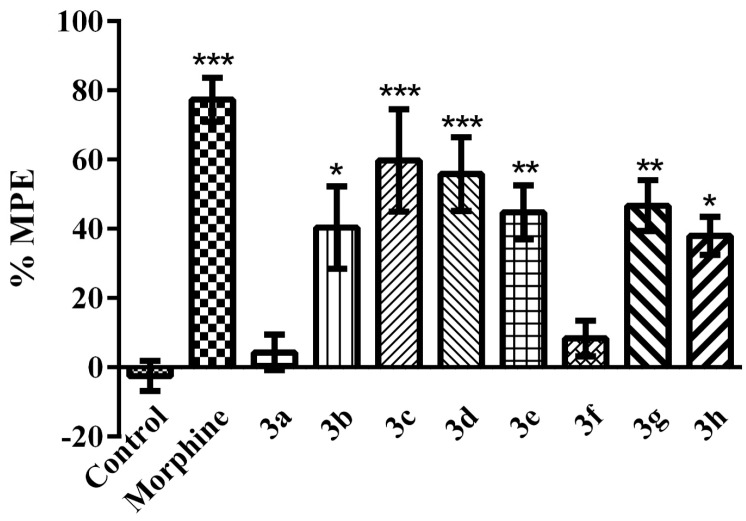
Effects of test compounds (100 mg/kg) and morphine (10 mg/kg) on maximum possible effect (MPE %) values of mice in the tail-clip test. Significance against control group * *p* < 0.05, ** *p* < 0.01, *** *p* < 0.001. Values are given as mean ± SEM. One-way ANOVA *post-hoc* Tukey’s test, *n* = 7.

As displayed in Figure 2, in tail-clip tests, reference drug morphine and compounds **3b**, **3c**, **3d**, **3e**, **3g** and **3h** caused prolongation in the response latencies of mice and induced a significant increase in the maximum possible effect % (MPE %) values, with respect to the control values (F (9, 60) = 9.28; *p* < 0.001).

**Figure 3:**
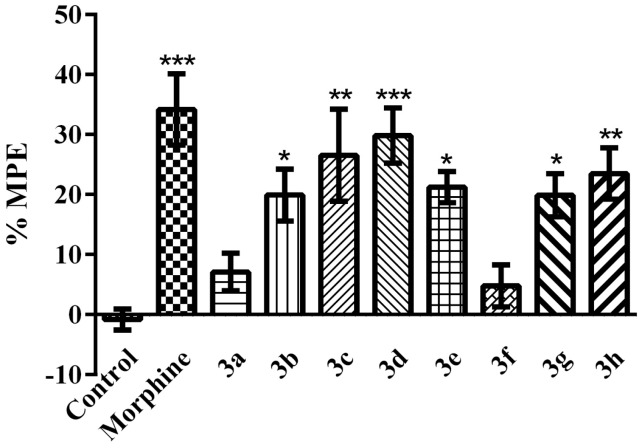
Effects of test compounds (100 mg/kg) and morphine (10 mg/kg) on maximum possible effect (MPE %) values of mice in the hot-plate test. Significance against control group * *p* < 0.05, ** *p* < 0.01, *** *p* < 0.001. Values are given as mean ± SEM. One-way ANOVA *post-hoc* Tukey’s test, *n* = 7.

As shown in Figure 3, in hot-plate tests, reference drug morphine and compounds **3b**, **3c**, **3d**, **3e**, **3g** and **3h** caused elongation in the response latencies of mice and induced a significant increase in the MPE % values, when compared to the control values (F (9, 60) = 6.58; *p* < 0.001).

**Figure 4:**
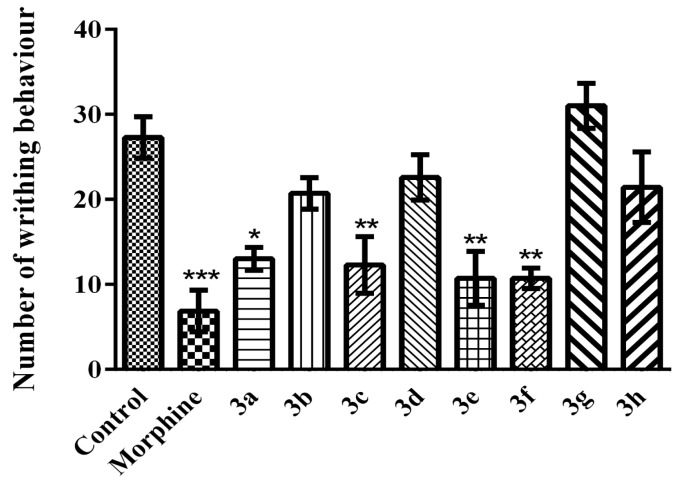
Effects of test compounds (100 mg/kg) and morphine (10 mg/kg) on number of writhing behavior of mice in the acetic acid-induced writhing test. Significance against control group * *p* < 0.05, ** *p* < 0.01, *** *p* < 0.001. Values are given as mean ± SEM. One-way ANOVA *post-hoc* Tukey’s test, *n* = 7.

In the acetic acid-induced writhing test, reference drug morphine and compounds **3a**, **3c**, **3e** and **3f** significantly reduced acetic acid-induced writhing responses of mice, with respect to the control values (F (9, 59) = 8.98; *p* < 0.001) (Figure 4).

**Figure 5:**
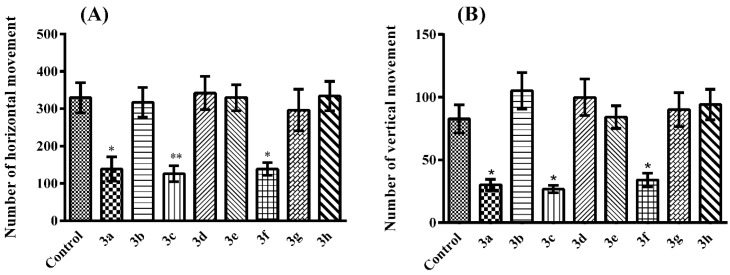
Effects of test compounds (100 mg/kg) on number of (**a**) horizontal and (**b**) vertical activity of mice in the activity cage test. Significance against control group * *p* < 0.05, ** *p* < 0.01. Values are given as mean ± SEM. One-way ANOVA *post-hoc* Tukey’s test, *n* = 7.

Following the administrations, compounds **3a**, **3c** and **3f** significantly decreased both horizontal (F (8, 54) = 6.48; *p* < 0.001) and vertical (F (8, 54) = 9.07; *p* < 0.001) locomotor activities of mice, in the activity cage measurements (Figure 5).

## 3. Discussion

In the present study, compounds **3a**–**h** were investigated for their probable antinociceptive effects on nociceptive pathways of nervous system. The effects of these compounds on mechanical, thermal and chemical stimuli were evaluated by tail-clip, hot-plate and acetic acid-induced writhing tests, respectively. In addition, an activity cage test was performed to assess the locomotor activity of animals.

A tail-clip test is a frequently preferred method for basic pain studies. The basis of this method is measuring the reaction time of rodents that feel constant mechanical pressure on their tails. In this study, the findings obtained from the tail-clip tests exhibited significant increases in the reaction time of mice following the administrations of compounds **3b**, **3c**, **3d**, **3e**, **3g** and **3h** (Figure 2). The results indicated the antinociceptive effects of these compounds on nociceptive pathways carrying noxious mechanical stimuli. These findings also suggest the possible localization of the antinociceptive activity in the medulla spinalis, since tail-clip tests mainly measure spinal reflexes [22,23]. Alterations in synaptic transmission of nociceptive input to the dorsal horn, modification in nociceptive processing in the spinal cord, or changes in spinal sensitization may be some of the possible mechanisms underlying the spinally mediated antinociceptive effects of compounds **3b**, **3c**, **3d**, **3e**, **3g** and **3h**.

A hot-plate test was performed in order to evaluate antinociceptive potential of the compounds against thermal noxious stimuli. In this test, thermal pain reflexes due to footpad contact with a hot surface were assessed. Licking is a rapid reaction to noxious thermal stimuli that is an indicator of nociceptive threshold, whereas jumping describes a more complex reaction, with a latency, and includes an emotional part of avoidance [24]. In the present study, the results of the hot-plate tests showed that the administration of compounds **3b**, **3c**, **3d**, **3e**, **3g** and **3h** significantly increased the reaction latencies of animals in response to constant exposure to heat (Figure 3), which indicated that the tested compounds acted on thermal nociceptive pathways. Antinociceptive effects observed in the hot-plate test seem to be related to supraspinal mechanisms, since this test predominantly measures supraspinally organized responses [22,23]. Modified transmission in the projections of nociceptive spinal cord neurons ascending to supraspinal sites, changes in supra-spinal sensitization, or alterations in the descending inhibitory mechanisms may be some of the possible mechanisms underlying the supraspinally mediated antinociceptive activities observed in this study.

The antinociceptive effects of the compounds on chemical noxious stimuli were examined by acetic acid-induced writhing test. In this inflammatory/irritative pain model, *i.p.* administration of acetic acid causes a reaction characterized by contraction of the abdominal muscles with simultaneous extension of the hind limbs and elongation of the body. Acetic acid is known to cause the release of several mediators (neurotransmitters/neuromodulators, kinins, histamine, acetylcholine, substance P, and prostaglandins, etc.), reducing the threshold of the nociception and stimulating the nociceptive neurons [24,25]. In this study, the data obtained from the acetic acid-induced writhing test showed that compounds **3a**, **3c**, **3e** and **3f** significantly reduced acetic acid-induced writhing responses of mice (Figure 4). The reduction in the number of writhes after the administration of these compounds suggests that the mechanism of compounds **3a**, **3c**, **3e** and **3f** may be related with a decrease in the release of inflammatory mediators in peripheral tissues or by direct blockage of its receptors, which results in antinociceptive activity. Furthermore, this activity could be produced by enhancement of the nociceptive threshold or by interruption of the transmission of the pain stimulus to the nerve fibre [25].

In the activity cage test, notable decreases were observed in the horizontal and vertical locomotor activities of mice following the administration of compounds **3a**, **3c** and **3f** (Figure 5). These findings indicated that the antinociceptive behavior of these three compounds might be caused by possible sedative effect or motor impairments. In other words, antinociceptive effects induced by compounds **3a**, **3c** and **3f** may not be specific.

Absence of deaths or unwanted adverse effects such as paralysis, ataxia, convulsions, and diarrhea pointed out the negligible toxicity of the tested compounds [24]. Nevertheless, the probable side effects of these compounds should be clarified with further detailed studies.

## 4. Materials and Methods

### 4.1. Chemistry

All reagents were purchased from commercial suppliers and were used without further purification. Melting points (M.p.) were determined on an Electrothermal 9100 melting point apparatus (Weiss-Gallenkamp, Loughborough, UK) and are uncorrected. IR spectra were recorded on an IRPrestige-21 Fourier Transform Infrared spectrophotometer (Shimadzu, Tokyo, Japan). ^1^H-NMR and ^13^C-NMR spectra were recorded on a Varian Mercury-400 FT-NMR spectrometer (Agilent, Palo Alto, CA, USA). Chemical shifts were expressed in parts per million (ppm) and tetramethylsilane was used as an internal standard. Mass spectra were recorded on an Agilent LC-MSD-Trap-SL Mass spectrometer (Agilent Technologies, Palo Alto, CA, USA). Elemental analyses were performed on a Perkin Elmer EAL 240 elemental analyzer (Perkin-Elmer, Norwalk, CT, USA).

#### General Procedure for the Synthesis of the Compounds

*4-(4-Nitrophenyl)thiosemicarbazide* (**1**) 4-(4-Nitrophenyl)thiosemicarbazide (**1**) was synthesized *via* the reaction of 4-nitrophenyl isothiocyanate (0.1 mol) with hydrazine hydrate (0.2 mol) as previously reported [26].

*5-(4-Nitrophenyl)amino-1,3,4-thiadiazole-2(3H)-thione* (**2**) [27] 4-(4-Nitrophenyl)thiosemicarbazide (**1**) was dissolved in a solution of potassium hydroxide in ethanol. Carbon disulfide was then added while stirring and the reaction mixture was heated under reflux for 10 h. The solution was cooled and acidified to pH 4-5 with hydrochloric acid solution and crystallized from ethanol.

*N-(Alkyl/Aryl)-2-[(5-((4-nitrophenyl)amino)-1,3,4-thiadiazol-2-yl)thio]acetamide derivatives* (**3a**–**h**). A mixture of *N*-(alkyl/aryl)-2-chloroacetamide/4-(chloroacetyl)morpholine (2 mmol) and 5-(4-nitrophenyl)amino-1,3,4-thiadiazole-2(3*H*)-thione (**2**) (2 mmol) in acetone was stirred at room temperature for 8 h in the presence of potassium carbonate. The reaction mixture was filtered. The residue was washed with water and crystallized from ethanol.

*N,N-Diethyl-2-[(5-((4-nitrophenyl)amino)-1,3,4-thiadiazol-2-yl)thio]acetamide* (**3a**) IR ν_max_ (cm^−1^): 3271, 3223 (N-H stretching), 2978, 2936, 2916, 2845, 2803 (Aliphatic C-H stretching), 1653 (C=O stretching), 1628, 1586, 1505, 1497 (N-H bending, C=N, C=C stretching and NO_2_ asymmetric stretching). ^1^H-NMR (400 MHz, DMSO-*d*_6_) δ (ppm): 1.04 (t, *J =* 7.2 Hz, 6.8 Hz, 3H, CH_3_), 1.17 (t, *J =* 7.2 Hz, 6.8 Hz, 3H, CH_3_), 3.28–3.41 (m, 4H, N-CH_2_), 4.35 (s, 2H, S-CH_2_), 7.79 (d, *J =* 9.2 Hz, 2H, aromatic), 8.25 (d, *J =* 9.2 Hz, 2H, aromatic), 11.09 (s, 1H, N-H). ^13^C-NMR (100 MHz, DMSO-*d*_6_): 12.89 (CH_3_), 14.05 (CH_3_), 37.40 (CH_2_), 40.03 (CH_2_), 41.88 (CH_2_), 116.88 (2CH), 125.50 (2CH), 140.83 (C), 146.05 (C), 155.69 (C), 163.56 (C), 165.44 (C). MS (ESI) (*m*/*z*): [M + H]^+^ 368.4. For C_14_H_17_N_5_O_3_S_2_ Calculated: C, 45.76; H, 4.66; N, 19.06. Found: C, 45.75; H, 4.64; N, 19.06.

*N-(3-Chlorophenyl)-2-[(5-((4-nitrophenyl)amino)-1,3,4-thiadiazol-2-yl)thio]acetamide* (**3b**) IR ν_max_ (cm^−1^): 3258, 3212 (N-H stretching), 3077, 3023 (Aromatic C-H stretching), 1672 (C=O stretching), 1596, 1568, 1539, 1505 (N-H bending, C=N, C=C and NO_2_ asymmetric stretching). ^1^H-NMR (400 MHz, DMSO-*d*_6_) δ (ppm): 4.21 (s, 2H, S-CH_2_), 7.14–7.16 (m, 1H, aromatic), 7.37 (t, *J =* 8.4 Hz, 7.6 Hz, 1H, aromatic), 7.44–7.47 (m, 1H, aromatic), 7.78–7.84 (m, 3H, aromatic), 8.23–8.26 (m, 2H, aromatic), 10.55 (s, 1H, N-H), 11.12 (s, 1H, N-H). ^13^C-NMR (100 MHz, DMSO-*d*_6_): 38.19 (CH_2_), 116.93 (2CH), 117.56 (CH), 118.64 (CH), 123.34 (CH), 125.46 (2CH), 130.54 (CH), 133.19 (C), 140.16 (C), 140.86 (C), 145.96 (C), 154.95 (C), 163.80 (C), 165.94 (C). MS (ESI) (*m*/*z*): [M + H]^+^ 422.8. For C_16_H_12_ClN_5_O_3_S_2_ Calculated: C, 45.55; H, 2.87; N, 16.60. Found: C, 45.54; H, 2.88; N, 16.61.

*N-(4-Chlorophenyl)-2-[(5-((4-nitrophenyl)amino)-1,3,4-thiadiazol-2-yl)thio]acetamide* (**3c**) IR ν_max_ (cm^−1^): 3271, 3221 (N-H stretching), 3165, 3098, 3030 (Aromatic C-H stretching), 2876 (Aliphatic C-H stretching), 1653 (C=O stretching), 1616, 1597, 1562, 1514, 1501, 1489 (N-H bending, C=N, C=C and NO_2_ asymmetric stretching). ^1^H-NMR (400 MHz, DMSO-*d*_6_) δ (ppm): 4.20 (s, 2H, S-CH_2_), 7.38–7.41 (m, 2H, aromatic), 7.61–7.64 (m, 2H, aromatic), 7.77–7.80 (m, 2H, aromatic), 8.23–8.26 (m, 2H, aromatic), 10.48 (s, 1H, N-H), 11.11 (s, 1H, N-H). ^13^C-NMR (100 MHz, DMSO-*d*_6_): 38.18 (CH_2_), 116.94 (2CH), 120.72 (2CH), 125.48 (2CH), 127.21 (C), 128.76 (2CH), 137.69 (C), 140.86 (C), 145.98 (C), 155.01 (C), 163.79 (C), 165.67 (C). MS (ESI) (*m*/*z*): [M + H]^+^ 422.8. For C_16_H_12_ClN_5_O_3_S_2_ Calculated: C, 45.55; H, 2.87; N, 16.60. Found: C, 45.55; H, 2.85; N, 16.62.

*N-(4-Nitrophenyl)-2-[(5-((4-nitrophenyl)amino)-1,3,4-thiadiazol-2-yl)thio]acetamide* (**3d**) IR ν_max_ (cm^−1^): 3343 (N-H stretching), 3078 (Aromatic C-H stretching), 2899, 2835, 2797 (Aliphatic C-H stretching), 1697 (C=O stretching), 1614, 1584, 1553, 1489 (N-H bending, C=N, C=C and NO_2_ asymmetric stretching). ^1^H-NMR (400 MHz, DMSO-*d*_6_) δ (ppm): 4.28 (s, 2H, S-CH_2_), 7.77–7.80 (m, 2H, aromatic), 7.84–7.87 (m, 2H, aromatic), 8.22–8.26 (m, 4H, aromatic), 10.97 (s, 1H, N-H), 11.14 (s, 1H, N-H). ^13^C-NMR (100 MHz, DMSO-*d*_6_): 38.24 (CH_2_), 116.86 (2CH), 118.86 (2CH), 124.90 (2CH), 125.30 (2CH), 140.86 (C), 142.44 (C), 144.73 (C), 145.90 (C), 154.70 (C), 163.77 (C), 166.48 (C). MS (ESI) (*m*/*z*): [M + H]^+^ 433.4. For C_16_H_12_N_6_O_5_S_2_ Calculated: C, 44.44; H, 2.80; N, 19.43. Found: C, 44.44; H, 2.81; N, 19.42.

*N-(1,3-Benzodioxol-5-ylmethyl)-2-[(5-((4-nitrophenyl)amino)-1,3,4-thiadiazol-2-yl)thio]acetamide* (**3e**) IR ν_max_ (cm^−1^): 3264, 3216 (N-H stretching), 3090 (Aromatic C-H stretching), 1654 (C=O stretching), 1641, 1620, 1599, 1578, 1518, 1501 (N-H bending, C=N, C=C and NO_2_ asymmetric stretching). ^1^H-NMR (400 MHz, DMSO-*d*_6_) δ (ppm): 3.98 (s, 2H, CH_2_), 4.19 (d, *J =* 5.6 Hz, 2H, CH_2_), 5.92 (s, 2H, O-CH_2_-O), 6.70 (d, *J =* 8.0 Hz, 1H, aromatic), 6.79 (d, *J =* 7.6 Hz, 2H, aromatic), 7.77 (d, *J =* 9.6 Hz, 2H, aromatic), 8.23 (d, *J =* 8.8 Hz, 2H, aromatic), 8.63–8.65 (m, 1H, N-H), 11.09 (s, 1H, N-H). ^13^C-NMR (100 MHz, DMSO-*d*_6_): 37.23 (CH_2_), 42.35 (CH_2_), 100.80 (CH_2_), 107.90 (CH), 107.94 (CH), 116.94 (2CH), 120.44 (CH), 125.49 (2CH), 132.79 (C), 140.86 (C), 146.05 (C), 146.09 (C), 147.26 (C), 155.21 (C), 163.74 (C), 166.60 (C). MS (ESI) (*m*/*z*): [M + H]^+^ 446.4. For C_18_H_15_N_5_O_5_S_2_ Calculated: C, 48.53; H, 3.39; N, 15.72. Found: C, 48.51; H, 3.40; N, 15.71.

*1-*(*Morpholin-4-yl)-2-[(5-((4-nitrophenyl)amino)-1,3,4-thiadiazol-2-yl)thio]ethan-1-one* (**3f**) IR ν_max_ (cm^−1^): 3271, 3225 (N-H stretching), 2963, 2916, 2845, 2806 (Aliphatic C-H stretching), 1654 (C=O stretching), 1630, 1589, 1512, 1501 (N-H bending, C=N, C=C and NO_2_ asymmetric stretching). ^1^H-NMR (400 MHz, DMSO-*d*_6_) δ (ppm): 3.43–3.61 (m, 8H, morpholine), 4.34 (s, 2H, S-CH_2_), 7.74–7.78 (m, 2H, aromatic), 8.21–8.23 (m, 2H, aromatic), 11.08 (s, 1H, N-H). ^13^C-NMR (100 MHz, DMSO-*d*_6_): 37.09 (CH_2_), 42.05 (CH_2_), 45.87 (CH_2_), 65.93 (2CH_2_), 116.86 (2CH), 125.44 (2CH), 140.83 (C), 145.99 (C), 155.18 (C), 163.70 (C), 165.30 (C). MS (ESI) (*m*/*z*): [M + H]^+^ 382.4. For C_14_H_15_N_5_O_4_S_2_ Calculated: C, 44.09; H, 3.96; N, 18.36. Found: C, 44.10; H, 3.97; N, 18.35.

*N-(Benzothiazol-2-yl)-2-[(5-((4-nitrophenyl)amino)-1,3,4-thiadiazol-2-yl)thio]acetamide* (**3g**) IR ν_max_ (cm^−1^): 3206 (N-H stretching), 3159, 3078, 3069, 3059 (Aromatic C-H stretching), 2957, 2934, 2914, 2843, 2805, 2725 (Aliphatic C-H stretching), 1684 (C=O stretching), 1613, 1597, 1568, 1506 (N-H bending, C=N, C=C stretching and NO_2_ asymmetric stretching). ^1^H-NMR (400 MHz, DMSO-*d*_6_) δ (ppm): 4.37 (s, 2H, S-CH_2_), 7.31–7.35 (m, 1H, aromatic), 7.44–7.48 (m, 1H, aromatic), 7.76–7.79 (m, 3H, aromatic), 8.00 (d, *J =* 8.0 Hz, 1H, aromatic), 8.21–8.25 (m, 2H, aromatic), 11.14 (br, 1H, N-H), 12.72 (br, 1H, N-H). ^13^C-NMR (100 MHz, DMSO-*d*_6_): 36.98 (CH_2_), 116.97 (2CH), 120.67 (CH), 121.74 (CH), 123.72 (CH), 125.46 (2CH), 126.21 (CH), 131.45 (C), 140.89 (C), 145.92 (C), 148.48 (C), 154.53 (C), 157.69 (C), 163.88 (C), 166.99 (C). MS (ESI) (*m*/*z*): [M + H]^+^ 445.5. For C_17_H_12_N_6_O_3_S_3_ Calculated: C, 45.94; H, 2.72; N, 18.91. Found: C, 45.95; H, 2.73; N, 18.90.

*N-(6-Nitrobenzothiazol-2-yl)-2-[(5-((4-nitrophenyl)amino)-1,3,4-thiadiazol-2-yl)thio]acetamide* (**3h**) IR ν_max_ (cm^−1^): 3298, 3204 (N-H stretching), 3152, 3084 (Aromatic C-H stretching), 2930, 2911 (Aliphatic C-H stretching), 1713, 1688 (C=O stretching), 1613, 1597, 1559, 1505 (N-H bending, C=N, C=C and NO_2_ asymmetric stretching). ^1^H-NMR (400 MHz, DMSO-*d*_6_) δ (ppm): 4.41 (s, 2H, S-CH_2_), 7.59 (d, *J* = 9.2 Hz, 1H, aromatic), 7.78 (d, *J* = 9.2 Hz, 1H, aromatic), 7.91–7.94 (m, 1H, aromatic), 8.21–8.30 (m, 3H, aromatic), 9.07 (s, 1H, aromatic), 10.96 and 11.21 (2s, 1H, N-H), 13.15 and 13.97 (2s, 1H, N-H). ^13^C-NMR (100 MHz, DMSO-*d*_6_): 36.95 (CH_2_), 116.88 (2CH), 118.91 (d, CH), 120.69 (d, CH), 121.68 (CH), 125.29 (2CH), 132.13 (C), 140.86 (C), 143.07 (C), 145.87 (C), 153.25 (C), 154.24 (C), 163.09 (C), 163.87 (C), 167.69 (C). MS (ESI) (*m*/*z*): [M + H]^+^ 490.5. For C_17_H_11_N_7_O_5_S_3_ Calculated: C, 41.71; H, 2.27; N, 20.03. Found: C, 41.70; H, 2.26; N, 20.02.

### 4.2. Pharmacology

#### 4.2.1. Animals

Adult BALB/c mice (30–35 g) were used for all tests. Animals were maintained under a 12 h light/dark cycle in a temperature (24 ± 1 °C) and humidity controlled room. To warrant adaptation to the new environment, mice were housed in the laboratory at least 48 h before the experimental session. Food was withdrawn 12 h before experiments in order to avoid food interference with substance absorption, though water was allowed ad libitum. The Local Ethical Committee on Animal Experimentation of Anadolu University, Eskişehir, Turkey has approved the experimental protocols.

#### 4.2.2. Administration of the Compounds

Mice assigned randomly to following experimental groups: control group (sunflower oil), reference drug (morphine sulfate, Sigma-Aldrich, St. Louis, MO, USA, 10 mg/kg, *i.p*) group and test compounds (100 mg/kg, *i.p*) groups. Each group consists of seven animals. Injections were administrated *(i.p*) 0.5 h before the tests.

#### 4.2.3. Assessment of Pain Behavior

##### Mechanical Nociceptive Test

The mechanical antinociceptive activities of the test compounds were evaluated by the tail-clip test in mice. A metal artery clamp was applied to the tail of mouse and the time spent before biting the clamp (response latency) was recorded by a stopwatch [28]. Elongation of response latency is considered as an antinociception parameter. A sensitivity test was carried out before the experimental session and animals that did not respond to the clamp within 10 s were discarded from the experiments. Maximum latency time (cut-off time) for the tail-clip tests was chosen as 10 s to avoid possible tissue damage [25].

##### Thermal Nociceptive Test

The thermal antinociceptive activities of the test compounds were evaluated by the hot-plate test in mice. Mice were placed on a hot-plate analgesiameter (Ugo Basile, Italy, No. 7280), which was set at 55 ± 1.0 °C and response latencies (time of licking the forepaws or eventually jumping) were recorded. Elongation of “response latency” is considered as an antinociception parameter. A sensitivity test was carried out before the experimental sessions and only the animals reacting within 15 s were selected for the tests. Maximum cut-off time was chosen as 30 s to prevent tissue damage [25].

Antinociceptive effect was calculated by converting the tail-clip and hot-plate latencies to percentage of the maximum possible effect (MPE %), according to the following equation [24]:

MPE % = [(post-drug latency − pre-drug latency)/(cut-off time − pre-drug latency)] × 10(1)

##### Chemical Nociceptive Test

The chemical antinociceptive activities of the test compounds were evaluated by the acetic-acid induced writhing test in mice [29]. Thirty minutes after the administrations, mice were treated with an aqueous solution of acetic acid (Merck, Rio de Janeiro, Brazil) (0.6% *v*/*v*, *i.p*) at a dose of 10 mL/kg to induce contractions. After 5 min, the number of abdominal constrictions and stretches during the following 10 min was recorded. Significant reduction in the number of writhes was considered as an antinociception parameter [30].

#### 4.2.4. Assessment of Spontaneous Locomotor Activity

##### Activity Cage Test

The spontaneous locomotor activities of mice were monitored by the activity cage apparatus (Ugo Basile, No.7420, Varese, Italy) containing two pairs of 16 photocells, 3 cm and 12 cm above the floor under a transparent cover. Interruptions of light beams to the photocells during vertical and horizontal movements of the animals were automatically recorded for 4 min [31].

#### 4.2.5. Statistical Analyses

Statistical evaluation was performed using GraphPad Prism (version 6.01 software; GraphPad, Inc., San Diego, CA, USA). The data used in statistical analyses were acquired from 7 animals for each group. Data obtained from the pharmacological tests were analyzed by a one-way ANOVA followed by a post hoc Tukey’s test. The results were expressed as mean ± standard error of mean (S.E.M). Differences between data sets were considered as significant when *p*-value was less than 0.05.

## 5. Conclusions

In the present paper, we described the synthesis of new 1,3,4-thiadiazole derivatives, which were evaluated for their antinociceptive activities.

Among the tested compounds, (1,3-benzodioxol-5-ylmethyl)amino substituted compound **3e** was the only compound that showed its specific antinociceptive activity by manipulating both central and peripheral nociceptive mechanisms. In addition, compounds **3b**, **3d**, **3g** and **3h** were shown to inhibit the transmission of nociceptive stimuli by affecting central nociceptive mechanisms without any interaction with peripheral pathways. These findings supported the previous papers reporting the centrally and/or peripherally mediated antinociceptive effects of 1,3,4-thiadiazole derivatives [11,12,13,14,21].

Since pain is a process concerning numerous neuronal signaling and modulatory pathways [32,33], various mechanisms (such as opioidergic, tachykininergic, cholecystokinergic, cannabinoidergic, cholinergic, monoaminergic, nitrergic systems or ion channels such as Ca, Na etc.) [34,35,36,37] might contribute to the antinociceptive effects of these compounds. Therefore, future studies are needed to clarify underlying mechanisms of the compounds.

## Figures and Tables

**Table 1:** The yields and melting points (M.p.) of compounds **3a**–**h**.

Compound	R	Yield (%)	M.p. (°C)
**3a**	Diethylamino	92	240.1
**3b**	(3-Chlorophenyl)amino	85	227.9
**3c**	(4-Chlorophenyl)amino	91	233.4
**3d**	(4-Nitrophenyl)amino	92	267.0
**3e**	(1,3-Benzodioxol-5-ylmethyl)amino	91	238.8
**3f**	Morpholin-4-yl	89	235.3
**3g**	(Benzothiazol-2-yl)amino	89	282.5
**3h**	(6-Nitrobenzothiazol-2-yl)amino	85	298.7
